# Analysis of PLA/PHB Biopolymer Material with Admixture of Hydroxyapatite and Tricalcium Phosphate for Clinical Use

**DOI:** 10.3390/polym14245357

**Published:** 2022-12-07

**Authors:** Miroslav Kohan, Samuel Lancoš, Marek Schnitzer, Jozef Živčák, Radovan Hudák

**Affiliations:** Department of Biomedical Engineering and Measurement, Faculty of Mechanical Engineering, Technical University of Košice, Letná 1/9, 04200 Kosice, Slovakia

**Keywords:** PLA/PHB + HA/TCP, filament, microscopic, cytotoxicity, mechanical testing

## Abstract

One trend in tissue engineering and regenerative medicine is the development of degradable composite polymers. The aim of this study was the comprehensive analysis of Polylactic acid (PLA)/Polyhydroxybutyrate (PHB) + Hydroxyapatite (HA)/Tricalcium phosphate (TCP) material from filament production to mechanical testing of samples with different infills and the production of an implant replacement for an intervertebral disc. Filament Maker—Composer 450 (3devo; Netherlands) was used to produce filaments. Experimental samples and the implant for the intervertebral disc were made using FDM technology using a DeltiQ2 3D printer (Trilab, Czech Republic). Mechanical testing of experimental samples was performed on an Inspekt TABLE 5 kN (Hegewald & Peschke, Nossen, Germany). Microscopic analysis, cytotoxicity test, and filament diameter analysis using descriptive statistics were also part of the focus. The results of the analysis of the diameter of the filament show that the filament meets the prescribed standard. The cytotoxicity test for PLA/PHB + HA/TCP material showed no toxicity. Microscopic analysis showed an even distribution of the ceramic component in the composite polymer. Mechanical testing showed a reduction in mechanical properties with 75% and 50% of the filling of experimental samples. All experimental samples subjected to mechanical testing showed higher tensile and compressive strength values compared to the values of the mechanical properties of vertebral trabecular bones, as reported in the literature. It can therefore be concluded that the material under investigation, PLA/PHB + HA/TCP appears to be a suitable candidate for hard tissue replacement.

## 1. Introduction

In today’s world, the development of new advanced materials plays a significant role in new applications in various industries. In connection with these progressive materials, the question is asked as to the selection of the appropriate technology for the actual implementation of the final output. According to statistical data in the study from the authors Wickramasinghe et al. [1], it is mentioned that additive manufacturing technologies are used in various sectors such as the automotive industry [2,3] or aerospace [4,5]. However, medicine is also a no less important sector where additive manufacturing technology is used [6,7,8]. Statistical data indicate that in 2018, additive manufacturing was used in this area at 0.973%. However, forecasts indicate that by 2026, its share of technology use will be as high as 18.2% [9]. This fact creates new questions and requirements for materials in the form of the production of new and high-quality filaments. Due to the fact that the materials PLA/PHB (polylactic acid / polyhydroxybutyrate) and also HA (Hydroxyapatite) and TCP (Tricalcium phosphate) have excellent properties within a biological environment, it can be concluded that this composite biopolymer is suitable for medical applications. This fact is confirmed by extensive studies examining various aspects of material research, whether chemical [10], morphological [11], production [12], biological [13], or mechanical [14].

One of the most common additive manufacturing technologies is Fused Deposition Modeling (FDM) [15]. This technology uses most commonly plastic strings (filaments) with a diameter of 1.75 mm or 2.85 mm. This raises new challenges for researchers and technologists to provide a well-made filament. A study by Kristiawan et al. [16] was intended to investigate the factors that influence the results of 3D printing in FDM technology. They agreed that filament production plays a key function in the 3D printing process, while the reduced quality of the 3D printing process, in turn, has an impact on the mechanical testing of experimental samples. Aspects that significantly reduce the quality of the filament are the composition of the filament, the working parameters of extrusion such as temperature, extrusion rate, and also the method of cooling the extruded filament. However, despite this, the author of the study states that there are certain relationships between factors and parameters that are not yet clear, and therefore in the future, it is very important to concentrate on optimization processes for composite materials, according to a review study by Wasti et al. [17] where FDM technology and its process parameters are described when using different composite polymers. The findings of this review study indicate that parameters such as layer thickness and height, infill application, extruder, and substrate temperature have an effect on the mechanical properties of the composite polymer. The study further describes that an important factor is also the choice of the polymer matrix, particle size and amount of filler, and homogeneity of the filament, which can affect the clogging of the extruder during the 3D printing process. A study by Koch et al. [18] describes an open-source hybrid 3D printer for the simultaneous printing of thermoplastics and hydrogels. The 3D printer enables alternating microextrusion of hydrogel and extrusion of molten thermoplastic fiber. Thanks to the 3D printer, two designs suitable for relevant bioprinting applications were created. The first design was a printed porous scaffold filled with hydrogel for bone replacement, and the second design was a printed plastic chamber that can be used in organ-on-a-chip applications. The advantage of this method in extruding two materials with two extruders is the reduction in demands on material characteristics in the form of mixing. However, from a structural point of view, it is necessary to carry out extensive modifications. A similar study by Lovecchio et al. [19] describes the modification of a commercial 3D printer based on the FDM principle with a 3D bioplotter. The goal of the work was to use these two devices and achieve the required porosity and thickness of the printed scaffold. The resulting prints of the given prototype showed sufficient resolution and repeatability, which indicates the given fusion between FDM technology and 3D Bioplotter technology is suitable for cell culture and tissue engineering. Another study by the author's Park et al. [20] describes the materials for the production of polymer-based filaments for additive manufacturing, where it explains the advantages/disadvantages of single and twin-screw extruders. Twin-screw extrusion was found to create a higher shear force between the material and the screw, which helps to better mix two or more components of the material and is, therefore, more suitable for the production of a composite polymer. Single-screw extrusion tends to produce insufficient shear deformation of the extruded material and, thereby, the accumulation of one component over another and poor mixing of the composite material. Therefore single-screw extrusion is suitable for single-component polymers.

Another area of research in composite biopolymer materials is microscopic analysis [21,22,23]. A study by Parias et al. [24] on the composite biopolymer PLA/PHB in multiple recycling examines the issue using SEM (Scanning Electron Microscope) analysis. This showed that with an increase in recycling cycles of more than 3 times, the course PHB particles in the PLA matrix became finer and more homogeneous. However, a greater number of recycling cycles also resulted in a significant reduction in viscosity. The author also noted that after multiple cycles, the percentage of crystallinity of the mixture as measured by DSC (Differential Scanning Calorimetry) increased from 6.3% to 17.5%.

Extensive studies on the subject of PLA/PHB material with an admixture of a ceramic component are also of interest [25,26,27]. A study by Senatov et al. [28] examines the mechanical properties of the PHB material with different percentages of HA ceramics (10%, 20%, 30%, 40%, and 50% mass fraction). From each material option, a scaffold was made using sintering technology. A high volumetric proportion of pore coupling was achieved at sizes of 50 μm—500 μm. Subsequently, the material options were compared with pure PHB. The comparison evaluated tensile and compressive mechanical properties, biocompatibility, osseointegration, and cytotoxicity. The most comprehensive results were achieved by a scaffold with a 20 percent share of HA, whose Young’s modulus of elasticity reached values of 901 MPa (against 106 MPa). PHB/20%HA samples showed no significant level of cytotoxicity after 24 h. The results of MMSC (Multipotent mesenchymal stromal cells) proliferation against net PHB (31% ± 6.1% vs. 20% ± 5.7%; *p* = 0.039) underwent significant improvement. In the ceramic admixture option, increased bone tissue formation was observed, making PHB/20%HA a more suitable option for biomedical engineering. Another study focused on mechanical testing is from the author's Porter et al. [29] under the name of porous HA/PHB composites made using a new centrifugation method. In addition to mechanical testing, they examined porosity, HA/PHB volume fractions, and surface adhesion of the resulting HA/PHB composites.

The results showed a slight increase in strength for all composites. Conversely, the coated samples showed increased rigidity compared to pure HA samples (from 35 to 105 MPa). The authors report that the increased rigidity in the coated samples was due to strong interactions between the HA and PHB phases.

The aim of the present study was to create a comprehensive study for PLA/PHB + HA/TCP material with real outputs in the form of manufactured filament and an implant for an intervertebral disc. The analysis was performed to examine the diameter of the filament produced to optimize the 3D printing process for the material. Further, mechanical testing under tensile and compressive conditions was performed. 

## 2. Methodology

### 2.1. Characteristics of the Material

For the production of filaments, a composite biocompatible material (Corbion, Amsterdam, The Netherlands) was chosen with a majority polylactic acid (PLA) content in a weight ratio of 85%. In order to improve biocompatibility, the biodegradable polymer Polyhydroxybutyrate (PHB) was added at 15% by mass. Tricytyl 2-Acetyl Citrate (TAC) was added to the composite mixture as a natural plasticizer due to its better workability in the 3D printing process at a mass fraction of 5%. In order to ensure cellular adhesion, the mixture was enriched with bioactive fillers in the form of Hydroxyapatite (HA) and β-Tricalcium phosphate (TCP) in a mass ratio of 10%. The mixing of these materials was performed using extrusion, while the composite material itself was pellet-shaped. Table 1 shows the basic chemical and physical properties of the investigated materials. Glass transition of HA and β-TCP was not analyzed (n.a.).

### 2.2. Filament Production

**Figure 1:**
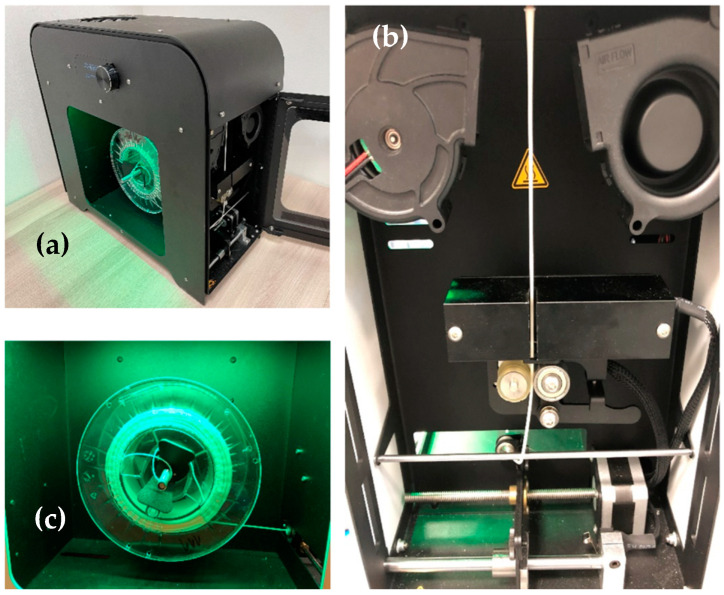
Production and measurement of manufactured filament from PLA/PHB + HA/TCP material. (**a**) Filament Maker, (**b**) Material extrusion, (**c**) Winding the filament onto the coil.

Before the production of the filament, the pellets were subjected to a drying process due to the possible appearance of moisture. For this purpose, an AirID Polymer Dryer (3devo, Utrecht, The Netherlands) was used. Drying took place at a temperature of 80 °C over a duration of 3 h. The entire filament production process was performed in an air-conditioned environment at a temperature of 18 °C. The production of filaments was performed using the Filament Maker—Composer 450 (3devo, Utrecht, Netherlands). The device includes 4 heating zones that melt the material. The temperature range of the heating zones was from 150 to 180 °C when the screw mechanism rotated 3 ± 0.4 RPM. The cooling of the extruded filament was performed using two fans. During the extrusion of the material in the form of a filament, the diameter of the filament was also measured using an optical sensor with an accuracy of 43 μm (see Figure 1). The sampling frequency was 1 s. The duration of the recording was 6000 s (1 h and 40 min). The boundary point range for measuring the filament diameter was set in the range of 1850 μm to 1650 μm. The cooling of the manufactured filament was performed using 2 fans located under the nozzle of the device. The nominal diameter of the filament was determined at 1750 μm. After the extrusion process, the newly formed filament was automatically wound onto a plastic spool.

### 2.3. Microscopic Analysis

For the purpose of expert analysis, an experimental technique of light microscopy was used: an inverse metallographic microscope (Olympus GX71, Tokyo, Japan) with a camera (Olympus DP12, Tokyo, Japan). The microscope used accessories for observation in polarized light and using differential interference contrast. The total number of samples for microscopic analysis was *n* = 10. The samples were randomly selected with different lengths of filament produced. The sample size was a pellet from filament with dimensions h = 3 mm; Ø = 1.75 mm. The samples were cleaned with methanol in an ultrasound before observation. The details of the microstructure were checked using scanning electron microscopy JSM 7000F (Jeol, Tokyo, Japan) in secondary electron mode (SEI), which obtained information on the morphology of ceramic particles and their distribution in the polymer filament matrix. The back-scattered electron (BSE) mode provided information about the distribution of elements in the sample by atomic number. The areas in which elements with a higher atomic number are present are lighter in this representation, and the areas formed by elements with a low atomic number are shown as darker. A necessary condition for the analysis of filament samples from the polymer matrix was to provide an electrically conductive surface of the preparation with each polymer matrix sample. Before being observed in an electron microscope, a layer of gold was deposited on all the samples analyzed. As a result, it was not possible to detect phosphorus in energy-dispersive X-ray (EDX) microanalyses. The K-alpha emission line of that overlaps with the M-alpha line of gold. The advanced preparation of samples for microstructure control involved preparation in dentacryle, sanding on sandpapers of granularity of 240, 400, 600, and 800, moistening with water, polishing with diamond paste, granularity 1/0 and 1/4 on satin moistened with kerosene, washing and rinsing with Ethanolum benzino denaturatum. 

### 2.4. Cytotoxicity Test

**Figure 2:**
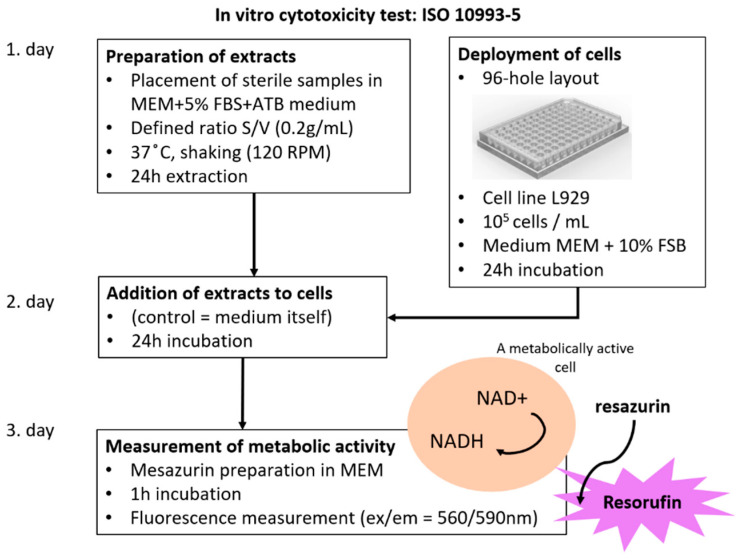
Details of the cytotoxicity test procedure.

The aim of the test was to determine the quantitative level of biocompatibility of the filament produced when performing an in vitro cytotoxicity test. The total number of pellet samples was *n* = 10, and they were randomly selected from the filament produced. The preparation of the samples was performed by inserting sterile samples into the minimum essential medium (MEM) + 5% FBS + ATB (acetyltheventin B). The defined ratio was S/V (0.2 g/mL). The temperature, in this case, was 37 °C with shaking at 120 RPM. The extraction was set at 24 h. The deployment of cells was performed through a 96-hole arrangement. The L929 cell line (1 × 10^5^ cells mL^−1^) was used. MEM + 10% FBS (Fetal bovine serum) was used as the medium. All these procedures were performed in conformance with ISO 10993-5. Figure 2 shows the details of the cytotoxicity test procedure

### 2.5. Production of Experimental Samples

**Figure 3:**
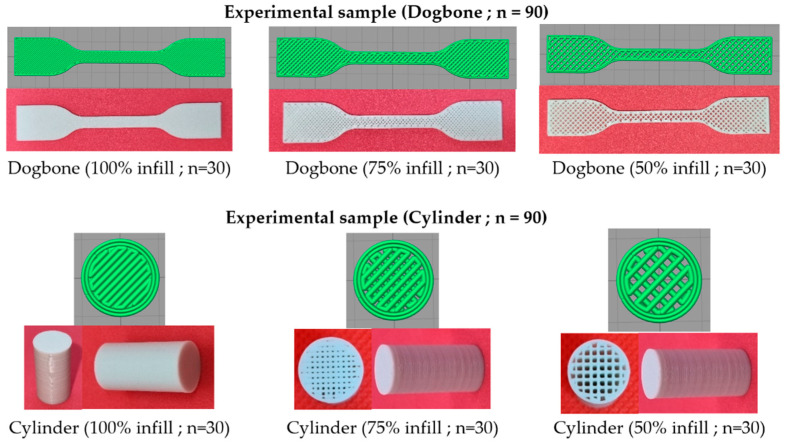
Preprocessing and printing experimental samples for mechanical testing (Cylinder—uniaxial mechanical compressive testing; Dogbone—uniaxial mechanical tensile testing).

A biocompatible composite filament of PLA/PHB + HA/TCP material was used for the production of experimental samples. The total number of samples produced was *n* = 180 for tensile and compressive mechanical testing. The first type of experimental sample was in a “Dogbone” shape, type 5B, according to the STN EN ISO 527-2 standard. The second type of experimental sample was a “Cylinder” shape with dimensions Ø = 10 mm, h = 20 mm. Both types of experimental samples were produced with 100%, 75%, and 50% infill (see Figure 3). Before the printing process, Simplify3D software was used for the correct positioning of the sample and also the parameter settings for 3D printing. For printing, a nozzle size of 0.4 mm was used to set the layer size to 0.25 mm and the layer thickness to 0.4 mm. The bed temperature was set at 65 °C and the extraction temperature at 220 °C. The cooling of the layers during printing was performed using a fan, which was part of the extruder. The print speed was set to 1800 mm/min. A DeltiQ2 3D printer (Trilab, Hradec Králové, Czech Republic) was used for printing. The temperature of the printing environment was 20 °C.

**Figure 4:**
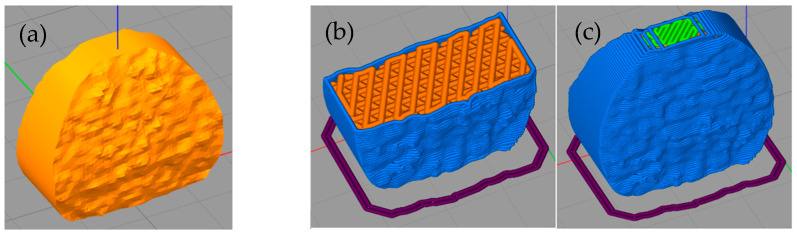
Pre-processing of 3D printing and output in the form of an intervertebral disc implant made of PLA/PHB + HA/TCP material. (**a**) Intervertebral disc model; (**b**) Simulation progress—detail on he filling of the model; (**c**) Final stage of simulation.

As part of the 3D printing process for PLA/PHB + HA/TCP material, an intervertebral disc implant was printed. The setup of the 3D printing process was the same as when printing experimental samples for mechanical testing. The design of the implant of the intervertebral disc was performed using SolidWorks software. The pre-processing of 3D printing was performed in Simplify3D software. The printing itself was performed using DeltiQ2 3D printer (Trilab, Hradec Králové, Czech Republic). The actual pre-processing can be seen in Figure 4. The simulation of the 3D printing process of the intervertebral disc model was performed in Simplify 3D software (Simplify 3D, Cincinnati, OH, USA). 

**Figure 5:**
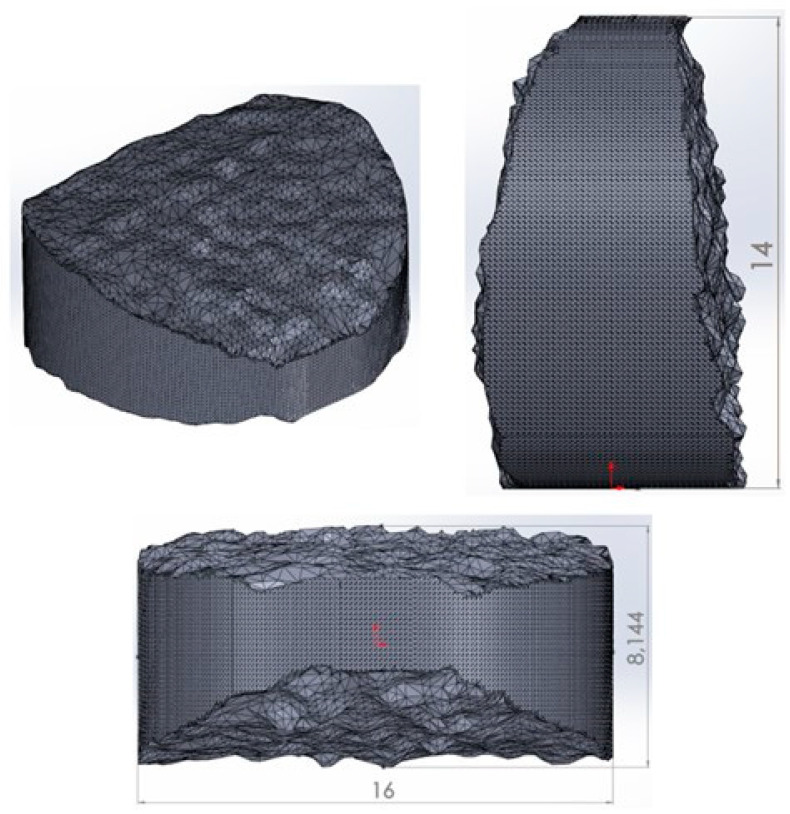
CAD model of the implant of the intervertebral disc (height, width, and thickness of the model).

The manufacture and design of the implant of the intervertebral disc from PLA/PHB + HA/TCP material also included a basic dimensional characteristic. The nominal values were considered to be those from the CAD model. The measurement of the CAD model was performed in SolidWorks software. The measurement of the printed implant was performed using an LM-1100 optical comparator (Keyence, Mechelen, Belgium). The accuracy of the device was determined at ±0.7 μm. The basic dimensions that were compared between the CAD model and the printed structure were the total height, overall width, and outermost points of the lateral and medial sides of the implant (see Figure 5).

### 2.6. Mechanical Testing

**Figure 6:**
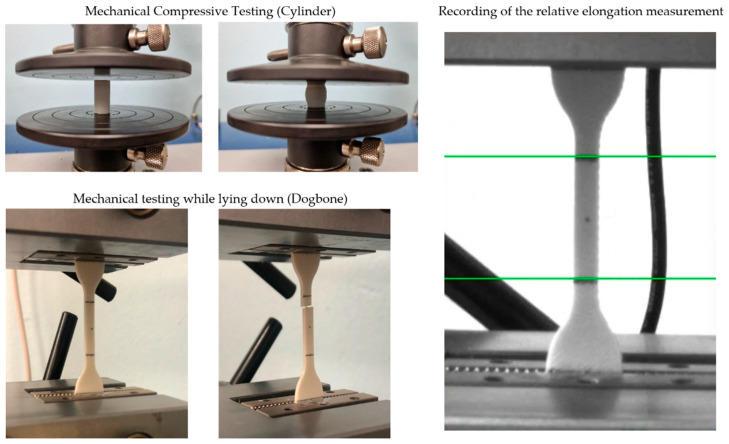
Mechanical tensile/compressive testing of experimental samples (Cylinder, Dogbone) and measurement of relative elongation.

The total number of experimental samples (*n* = 180) with different infills (100%, 75%, and 50%) were subjected to mechanical tests, with 90 samples being analyzed using a single-axis tensile test and 90 samples using a single-axis compressive test. Mechanical testing was performed on the Inspekt TABLE 5 kN (Hegewald & Peschke, Nossen, Germany). The set maximum power of the device was at 5 kN. Experimental samples were mechanically tested in accordance with STN EN ISO 527-2:2012. The size of the experimental samples was in the form of a “Dogbone” type 5B and a cylinder in dimension Ø = 10 mm, h = 20 mm. The loading speed was set at 2 mm/min. The distance of the clamping mechanism was given at 50 mm. The RTSS extensometer (LIMESS, Krefeld, Germany) was used to detect relative elongation. The tested area of the experimental sample examined in mechanical tensile testing was 20 mm, according to the applicable standards. In Figure 6, it is possible to see the fixation of experimental samples in mechanical tensile/compressive testing and also the detection of relative elongation using the extensometer.

## 3. Results and Discussion

### 3.1. Analysis of Manufactured Filament

The diameter of the filament plays a significant role in the 3D printing process. The aim of the analysis is to determine the quality of the filament in relation to its diameter. Descriptive statistics were used for this analysis, comparing the nominal value of the filament diameter of 1750 μm. The parameters evaluated were the average diameter (the value expresses the mean level of the total set of measured values), MIN/MAX value (minimum/maximum measured value), standard deviation (a value that determines the statistical dispersion), variance (variability of the data in the set from the calculated average value) and skewness (expresses the skewness of the population and therefore whether values higher or lower than the average predominate in the given population).

Table 2 describes the analysis of the filament produced using descriptive statistics. By comparing the nominal value (1750 μm) with the measured average diameter (x) of the filament produced, a difference of 22.3 μm was found. The standard deviation, in this case, was ±22.3 μm. After recording these parameters, the tolerance margin of the filament produced is 44.6 μm. When comparing commercially produced filaments, manufacturers give a tolerance margin of ±50 μm [30,31], which means that the filament produced by us falls within the tolerance margin indicated by the commercial filament manufacturer. The maximum measured mean value was recorded in 1840 μm and the lowest at 1663 μm.

Another parameter for analyses of the filament produced is the VAR parameter, which determines the variability in the set and, therefore, how the measured values are different from the mean. This parameter, in this case, is −0.00049, which represents the minimum variance in the statistical population. When analyzing the SKS parameter, it was found that the value of the parameter is −0.1006, which means that the measured values of the diameter of the filament produced from PLA/PHB + HA/TCP material show higher values than the nominal value (1.75 mm) in the statistical set. This means that the filament produced is predominantly thicker than the set nominal value. 

A similar experience with the results of filament production was also recorded in a study by the authors Cardona et al. [31], where they describe the effects of filament diameter tolerance using FFF (Fused Filament Fabrication), and their research showed that manipulating both the speed and the height of the nozzle can affect the volumetric flow. This fact also correlates with our results, where when after changing the PRM even by small values, the diameter of the filament produced changed. However, in this case, the measured diameter of the filament is not the same throughout the measurement. This can be due to several factors, such as melt temperature [32,33,34], the rotational speed of the screw mechanism [35], cooling of the filament produced [36], or nozzle design [37].

**Figure 7:**
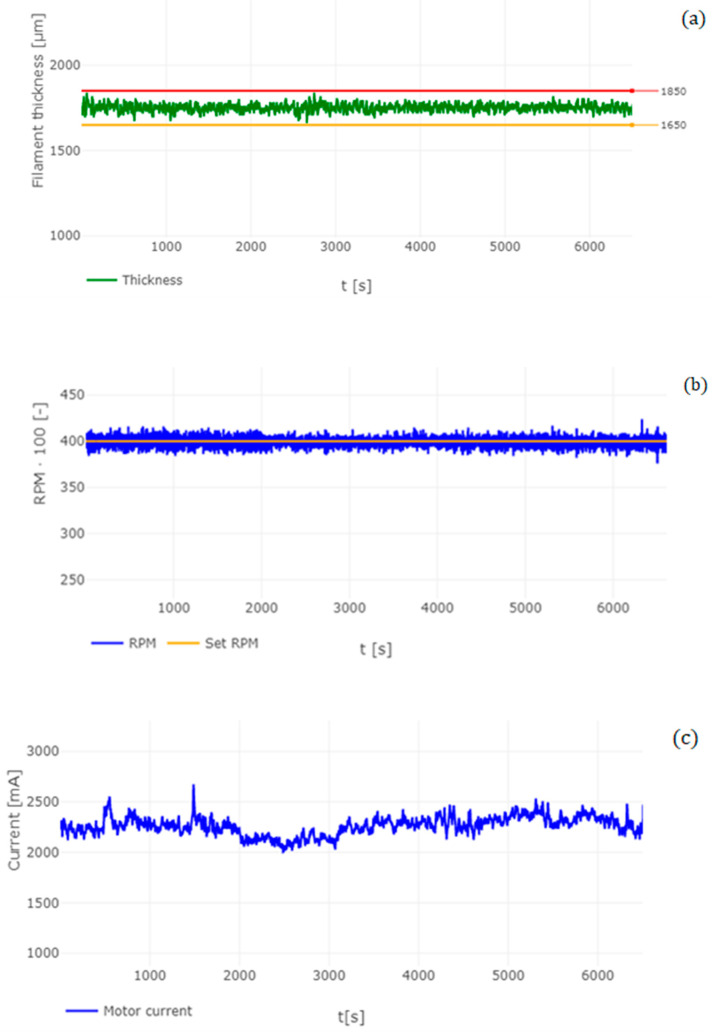
Analysis of filament produced (**a**), Measurement of the diameter of the filament; (**b**), measurement of RPM Filament Maker (**c**) Measurements of current on Filament Maker.

Figure 7a shows the measurement of the filament diameter during the manufacturing process. The figure also shows the limit values of the filament diameter (min = 1650 μm; max = 1850 μm). It is clear that in the total measuring range of the diameter of the filament produced, these limit values were not exceeded. Figure 7b shows changes in the level of current that was required to extrude the material during filament production. When comparing Figure 7a,b, it can be observed that over a time period of 1500 s, the current of the apparatus increases slightly, which may be due to the higher stress on the screw mechanism during the extrusion of the material. Subsequently, over the time period in Figure 7a, we can observe a slight increase in the diameter of the filament. This can be caused by a higher ceramic component in the melt resulting in a higher viscous factor which can affect filament production. This fact is confirmed by studies by the authors Chen et al. [38] and Mackay et al. [39].

A study by Phan et al. [40] describes the computational simulation of the fluid dynamics of the melting process in additive manufacturing using fusion fiber technology. The simulations show that in melting and shifting the melt, a recirculation vortex is formed, while the high viscosity in this vortex effectively seals the extrusion system, generating a voltage of around 10 MPa. This tension is the result of standard extrusion conditions with pure materials.

However, in our case, a multi-component material was used where one of the components is ceramics in the form of HA and TCP. Due to the fact that ceramic material has high thermal stability [41,42,43], it is highly likely that these particles in composite materials cause higher viscosity values than in pure PLA materials. For this reason, it can be concluded that the increased viscosity of the material during the extrusion process has an impact on the quality and, therefore, on the diameter of the filament produced, which also affects the 3D printing process itself [44].

### 3.2. Microscopic Analysis of the Filament Produced

**Figure 8:**
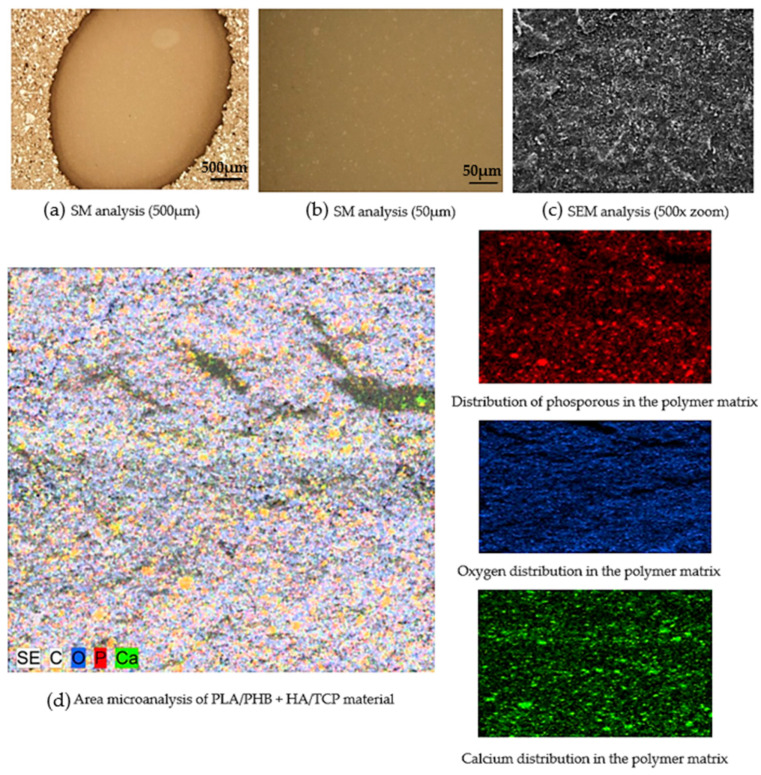
Microscopic analysis of composite biopolymer PLA/PHB + HA/TCP. (**a**) SM analysis (500 µm); (**b**) SM analysis (50 µm); (**c**) SEM analysis (500× zoom); (**d**) Area microanalysis of PLA/PHB + HA/TCP material.

Microscopic analysis plays a significant role in the evaluation of composite polymers. The purpose of this analysis was to quantitatively describe the distribution of the ceramic component in the composite biopolymer PLA/PHB. Figure 8 shows a stereo microscope (SM) image of a given composite polymer at a scale of 500 μm (Figure 8a) and 50 μm (Figure 8b). Figure 8a,b indicate that the PLA and PHB components show a high level of mixing. This is due to the molecular mass of the PLA and PHB polymers. While PLA has a high molecular mass (Mw > 18,000), PHB has a low molecular mass (Mw ˂ 18,000), and this caused the two components to mix well. Minor clusters of HA and TCP were noted in all observed samples. HA and also TCP is concentrated throughout the cross-section of the sample in small clusters. For all samples examined, a cluster size above 5 μm was not exceeded.

In the surface microanalysis of the PLA/PHB + HA/TCP material in the samples examined, a two to three-layer composite structure of the polymer matrix was observed. Defects in the integrity of the polymer matrix were not detected in all samples. Figure 8 shows the distribution of phosphorus, oxygen, and calcium elements. In all cases, the distribution was uniform except for small clusters that were not significant.

There are several studies that disassemble HA material in polymers based on SEM analysis. One of them is a study by Ramier et al. [45], namely biocomposite scaffolding for bone tissue applications. The scaffolds were prepared by electrospinning with a PHB/HA mixture and compared with scaffolds that had been coated with HA using electro-spraying. On the scaffolds using SEM analysis, the presence of an HA element on the outer surface was demonstrated. The clusters of HA grew primarily along the fibers, forming several nodes between the fibers in the direction of spraying. This fact correlates with our results. This is evidence of the appropriate application of the HA element. By using FDM technology and also the electrospinning technique, it is possible to produce samples that will have an even distribution of HA material over the entire surface or cross-section. 

Another similar study is from the authors Danoux et al. [46], where they evaluate the biologically active PLA/HA composite for bone regeneration. The percentage composition of this composite was 50/50. After 12 weeks of exposure to saline, SEM analysis revealed a dense surface area in PLA/HA samples, while samples from pure PLA showed a rather porous surface. This result is due to better degradation of pure PLA samples compared to samples with an admixture of HA. However, in cell proliferation, a higher value was recorded for samples from PLA/HA material. This evidence supports the theory that the HA component aids in better osteointegration between the bone and the composite biomaterial. Another study on this topic discussed the production and evaluation of biodegradable composites based on the PHB/PHV copolymer by a group of researchers Chen et al. [47]. As an admixture to the copolymer, they used ceramic components in the form of HA and TCP. Part of this investigation was also the analysis of the distribution of these ceramic components. The results point to a homogeneous distribution of HA and TCP in the PHB/PHV copolymer, even after pressing. These results correspond to the results of our study. This may also be due to the high level of pre-treatment of the materials studied. In our case, the materials in question were joined using extrusion techniques, and pellets were created, which were used in the production of filament. In the study by Chen et al., the starting materials were in the form of fine powders (for HA and TCP) and small granules (for PHB/PHV), which were mixed at 140 °C and 25 RPM. 

### 3.3. Test of Cytotoxicity

It is important to subject biomedical materials that are intended for medical application to biological tests to see if they are a potential risk to the human body. In this case, a significant role is played by the cytotoxicity test and cell proliferation on a given composite biopolymer in the form of PLA/PHB with the admixture of HA and TCP. 

**Figure 9:**
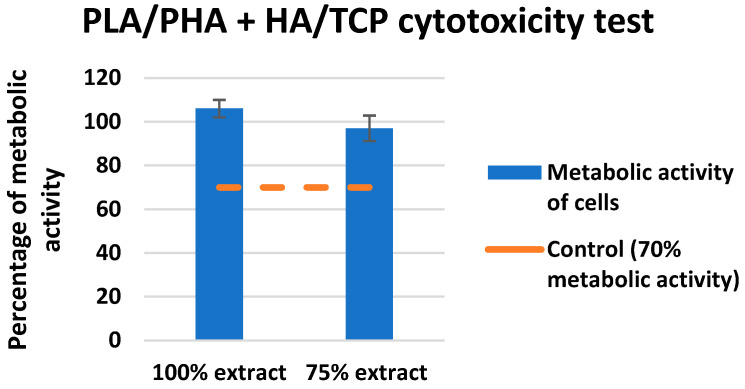
Cytotoxicity test for PLA/PHB + HA/TCP material using L929 fibroblast mouse.

**Figure 10:**
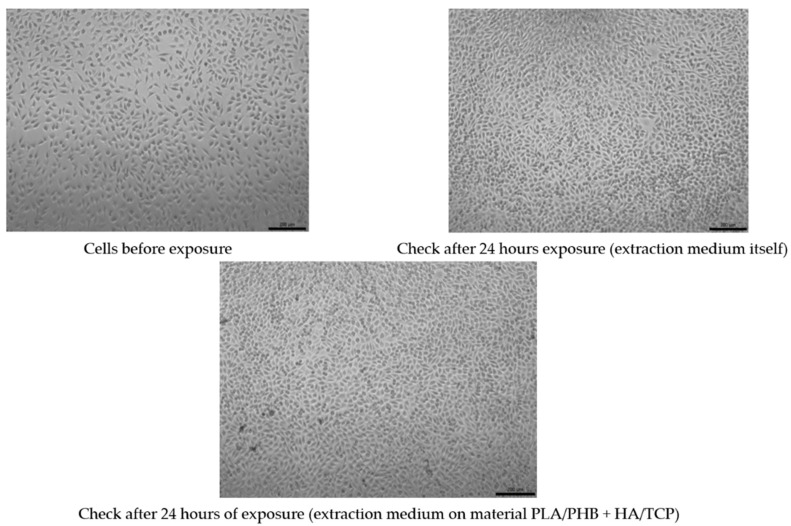
Quantitative imaging of cell culture (check vs. 24 hrs. exposure).

The evaluation of the metabolic activity of the cells on the investigated material can be seen in Figure 9. In Figure 10, quantitative results for the cytotoxicity of PLA/PHB + HA/TCP material can be observed when using mouse fibroblast L929. The control metabolic activity was set at 70%. 

None of the extracts of the test materials caused a decrease in the metabolic activity of cells below 70%, even after a negative control, even with the reference material. Only sample No. 4 showed slightly reduced metabolic activity (still above 70%). It follows from the above that the extracts are not cytotoxic. A visual inspection showed that in all types of material, there was an appropriate increase in the number of cells after 24 h of cultivation with the extract, which means proliferation. Cells after culture with extracts are morphologically comparable to unaffected cells, which means that the extracts are not cytotoxic. 

The results obtained correlate with the study by Kovalcik et al. [48], where they investigated the properties of a skeleton made of PLA and PHB material prepared using FDM technology. In the evaluation, the scaffolds were subjected to a cytotoxicity test with no negative results for toxicity. On the contrary, a large proliferation of mouse embryonic fibroblast cells was demonstrated within 96 h. However, there are also studies that describe materials that are physically modified for better cell growth, but this, in some cases, does not avoid changing the mechanical properties [49]. A similar study by Moorkoth et al. [50] investigated the cytotoxicity of biopolymer nanoparticles from PLA and PHB material using a cell viability test (MTT). The results from the authors show that no significant change in viability was detected in any of the tested concentrations, suggesting that the cells in question are viable in the presence of these types of materials.

### 3.4. Mechanical Testing Analysis

The aim of the analysis was to detect mechanical properties in uniaxial compressive/tensile testing for PLA/PHB + HA/TCP material with different infills of experimental samples. The parameters studied were Young’s modulus of elasticity (E), the yield point (Rp), and the maximum achieved force (Fmax). The student’s T-test was used to determine statistical significance. 

#### 3.4.1. Tensile Mechanical Testing

**Figure 11:**
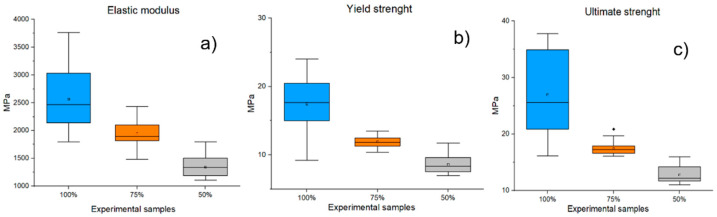
Results of mechanical tensile testing for PLA/PHB + HA/TCP material with infills of 100%, 75%, and 50% infills of experimental samples (**a**) Elastic modulus; (**b**) Yield point; (**c**) Ultimate strength).

Figure 11 shows the parameters E, Rp, and Fmax of single-axis tensile tests for PLA/PHB + HA/TCP material at 100%, 75%, and 50% infill of experimental samples. The mean E value for samples with 100% infill was recorded at 2566.78 ± 533.32 MPa, while the average value of samples with 75% infill was recorded at 1947.45 ± 220.9 MPa. Experimental samples with 50% infill have even lower values for this parameter (1345.86 ± 180.76 MPa). When comparing experimental samples with 100% and 75% infill, a decrease in E of 24.13% was observed, while for samples with 50% infill, this parameter was reduced by up to 47.57%. Similar results were also noted for the examined parameters Rp and Fmax. The mean Rp for samples with 100% infill was 17.45 ± 3.88 N/mm^2,^ while samples with 75% infill showed Rp at 11.81 ± 1.16 N/mm^2^. The Rp value for 50% infill samples was detected at 8.76 ± 1.48 N/mm^2^. By comparison, it was found that samples with 75% infill had lower Rp values than samples with 100% infill by 32.32%. A decrease in about 49.8% in the level of Rp was also observed for samples with 50% infill. The mean Fmax for samples with 100% inflation was found to be 26.97 ± 7.26 MPa, but for samples with 75% infill, 17.43 ± 1.19 MPa. A comparison of these two groups of samples revealed a decrease in Fmax of 35.37%. The average values of samples with 50% infill show a value of 12.79 ± 1.48 MPa, which means a 52.62% decrease in the parameter when compared to samples with 100% infill. The statistically significant (p ˂ 0.001) results were the comparisons of the sample groups with 100% and 75% infill and also with 50% infill for all the parameters studied (E, Rp, Fmax).

#### 3.4.2. Compressive Mechanical Testing

**Figure 12:**
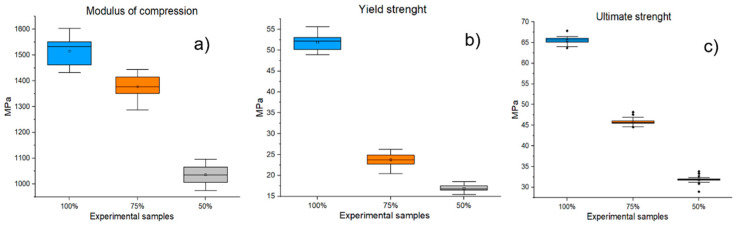
Results of mechanical tensile testing for PLA/PHB + HA/TCP material with infills of 100%, 75%, and 50% infills of experimental samples (**a**) Elastic modulus; (**b**) Modulus of compression; (**c**) Ultimate strength.

Figure 12 shows the investigated parameters (E, Rp, Fmax) of the mechanical compressive tests for the material PLA/PHB + HA/TCP at 100%, 75%, and 50% infill experimental samples. By comparing individual groups of experimental samples, the statistical significance (*p* ˂ 0.001) of the decrease in mechanical properties (E, Rp, Fmax) in samples with infills of 75% and 50% versus experimental samples with 100% infills was demonstrated. 

The mean “Modulus of compression” for samples with 100% infill was 1515.2 ± 50.97 MPa, while the average for 75% infill samples was 1377.62 ± 45.93 MPa. This difference represents a reduction in the Modulus of compression of 9.08%. Experimental samples with 50% infill showed an even greater decrease in this parameter (a decrease in 31.61%). In the evaluation of the Rp parameter, the mean values of samples with 100% infill were recorded at 51.89 ± 1.87 MPa, while the mean value for samples with 75% infill was 23.75 ± 1.4 MPa. The difference represents a decrease in the parameter by 54.19%. Experimental samples with 50% infill showed mean Rp values of 16.93 ± 0.74 MPa, which represents a decrease in the Rp parameter of 67.37% compared to samples with 100% infill. Similarly, decreasing results were also reported for the Fmax parameter (75% infill samples = 29.3% decrease; 50% infill samples = 51.42% decrease). 

The application of a new composite biopolymer for medical applications requires a detailed analysis of mechanical testing. Young’s modulus of elasticity (YME) plays a significant parameter in determining the appropriate application [51]. According to expert literary sources, this parameter is different for every single component (PLA = 4–5 GPA; PHB= 3–3.3 GPa; HA= 80–175 GPa; TCP= around 100 GPa) [52,53,54]. A similar study by Russias et al. [55] investigated the mechanical properties of composite materials at different grain sizes and HA concentrations in PLA coupling. They demonstrated that the modulus of elasticity of HA composites is heavily dependent on HA content. The largest YME value was recorded for an 80% HA mass fraction in the composite, with the value being measured as slightly below 10 GPa. This finding did not correspond to our results when the YME values measured by us averaged around 2.5 GPa. This significant difference in results could be due to a number of factors. One of them may be a different technological process for the production of samples [56]. Another reason for the difference in results may be due to the different mass ratios of the HA component in the composite, which is confirmed by a study by the authors Ingole et al. [57]. Another similar study, which focused on the mechanical properties of PHB composite material with different mass concentrations of HA, is described by the group of authors Tehrani et al. [58]. They found that they had the highest YME at a concentration with a 5% mass fraction of HA. This finding is partly correlated with our results. The partial difference in results can be attributed to the PLA component in our composite material, which shows higher YME values compared to the PHB material. An equally significant factor is that, in our case, the PLA material had a representation of up to 70% of the total mass ratio.

Another study focusing on the effects of combined fill patterns on mechanical properties in the FDM process is discussed by a group of researchers in the study by Dezaki et al. [59]. From the study, it can be concluded that the most critical parameters in terms of the strength properties of the printed structure are the layer thickness, the fill pattern, and the fill density. The density of the filler can reduce the YMP value several times. This finding correlates with our mechanical testing results for samples with 75% and 50% infill. The cause of the YME sample reduction in the cross-section area. Samples with 100% infill have a cross-section area of approximately 8 mm^2^, samples with 75% infill have a cross-section area of about 6 mm^2^, and samples with 50% infill have only 4 mm^2^. This fact is confirmed by a study by the authors Bakir et al. [60].

### 3.5. Analysis of the Dimensions of the Printed Implant

**Figure 13:**
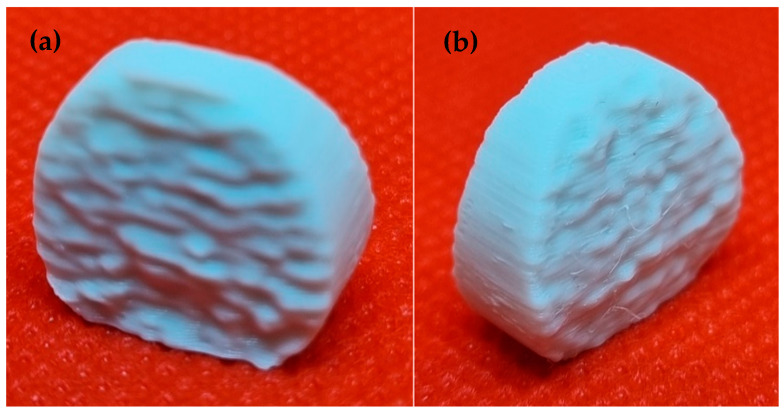
The implant of the intervertebral disc printed by FDM technology (**a**) Bottom side of the intervertebral disc model; (**b**) Top side of the intervertebral disc model.

The aim of the analysis of the basic dimensions of the printed implant of the intervertebral disc was to determine the dimensional characteristics of the CAD model as a reference value and the printed implant. Table 3 shows the nominal values of the dimensions examined on the CAD model and the measured values on the printed implant made of PLA/PHB + HA/TCP material. Figure 13 shows the printed implant of the intervertebral disc made from the studied material. 

When comparing the basic dimensions of the 3D model and the printed implant, minimal deviations were detected. In terms of the height of the structure studied, a difference of 33 μm was noted. When measuring the overall width of the implant, a deviation of 37 μm was noted. When measuring the total thickness of the implant, a difference of 0.054 μm was noted. 

The accuracy of 3D printing technology is essential for clinical applications. The accuracy of 3D printing is influenced by several factors [61,62]. A study by Kim et al. [63] describes the accuracy of 3D printing of surgical guides for dental applications. The aim of the study was to compare the dimensional differences between the CAD model and surgical guides in 3 types of 3D printers. Moreover, in this case, differences were noted between the CAD model and the printed surgical guides (0.06 ± 0.05 mm for PolyJet, 0.09 ± 0.05 mm for SLA, and 0.31 ± 0.33 mm for MJP). These results correspond to the results of our study. However, it should be noted that the equipment used, and the methodology for evaluating the measured data have a significant influence on the accuracy of printed structures. A similar study by the authors El-Katatny et al. [64] analyzed the defects of medical models produced by FDM technology. The study showed an absolute mean deviation of 0.24% for skull models and 0.22% for jaw models using ABS material. These results are partly correlated with our results, and in our study, we also noted variations, but not so significantly. This could be due to a number of factors. The first is that the model was modified in the Magics software, where there may have been a minor change from the original dimensions from the CT scan. The shrinkage of the material during 3D printing also plays an important role in this case. ABS is considered a polymer that has a high level of shrinkage (around 0.8% of the volume), while the PLA material has a low level of shrinkage, which precedes the deformation of the structure and, therefore, the accuracy of the printed structure. 

## 4. Conclusions

Comprehensive analysis and outputs in the form of manufactured filament and implant of the intervertebral disc from PLA/PHB + HA/TCP material indicate a promising future for medical applications. Analysis of the diameter of the filament showed that the filament produced meets the required standard for the 3D printing process in terms of diameter, which was confirmed by the printing itself. Microscopic analysis showed a homogeneous distribution of the ceramic component HA and TCP in a given filament. The cytotoxicity test did not show any toxic effects of the material, and, on the contrary, the level of cell proliferation exceeded the established standard. Mechanical testing of experimental samples with different fillings amply proves that mechanical properties are suitable to replace hard tissues.

## Figures and Tables

**Table 1:** Chemical and physical parameters of the materials used.

	PLA	PHB	HA	β-TCP
Melting point temperature [°C]	170–180	175–180	1100 °C	1670 °C
Glass transition temperature [°C]	50–80 °C	5 °C	n.a.	n.a.
Molecular weight [g/mol]	72.06	86	502.3	310.18
Density [g/cm^3^]	1.24	3.07	5.12	1.25
Granulate size	2–4 mm	2–4 mm	2–6 µm	2–6 µm
Morphology	spherical	spherical	angular	spherical/ angular
Purity	Medical grade	Medical grade	>95%	>95%
Supplier	Purasorb^®^ (Amsterdam, Netherlands)	Biomer P300 (Frankfurt, Germenny)	Captal^®^ (Pune, India)	Captal^®^ (Pune, India)

The individual components were mixed using a twin-screw extruder, while in the first phase, pellets of PLA/PHB materials were formed. Subsequently, this process was repeated with the same extrusion system but as another admixture, in this case, HA and TCP. As a result, pellets were created from the PLA/PHB + HA/TCP composite polymer.

**Table 2:** Analysis of filament produced using descriptive statistics.

**Filament Diameter (Average)**	**1.773 mm**
Standard deviation (SD)	0.0223 mm
Max. value of filament	1.84 mm
Min. value of filament	1.663 mm
Variance (VAR)	−0.00049
Skewness (SKS)	−0.1006

**Table 3:** Dimensional parameters of the CAD model (reference value) vs. dimensional parameters of the printed implant.

	3D Model (Nominal) [mm]	Printed Implant [mm]
Height	14	13.967
Width	16	15.963
Thickness	8.144	8.198

## Data Availability

Not applicable.
